# Supplementary material for: The role of uncertainty intolerance in adjusting to long-term physical health conditions: A systematic review
Source: PLoS One. 2023 Jun 2;18(6):e0286198. doi: 10.1371/journal.pone.0286198 (PMC10237456; doi:10.1371/journal.pone.0286198)
Supplement: S1 File — (DOCX) [file pone.0286198.s003.docx]

ORCID IDs

Benjamin Gibson: <https://orcid.org/0000-0002-9932-7403>

Benjamin Rosser: <https://orcid.org/0000-0002-1492-2706>

Jekaterina Schneider: <https://orcid.org/0000-0002-6069-4783>

Mark Forshaw: <https://orcid.org/0000-0001-8916-1633>
