# Supplementary material for: The role of uncertainty intolerance in adjusting to long-term physical health conditions: A systematic review
Source: PLoS One. 2023 Jun 2;18(6):e0286198. doi: 10.1371/journal.pone.0286198 (PMC10237456; doi:10.1371/journal.pone.0286198)
Supplement: S1 Table — (PDF) [file pone.0286198.s004.pdf]

Supporting information

S1 Table. Search Terms and Search Strategy.

Long-Term Physical Health Conditions	Search Terms
Terms related to intolerance of uncertainty	1. 'Intolerance of uncertainty'; OR for each term<br>2. 'Uncertainty intolerance'<br>3. 'Uncertainty tolerance'<br>4. 'Tolerance of uncertainty'<br>5. 'Intolerance of ambiguity'<br>6. 'Ambiguity intolerance'<br>7. 'Ambiguity tolerance'<br>8. 'Tolerance of ambiguity'
	AND
General terms related to long-term physical health conditions	9. 'Long-term condition'; OR for each term<br>10. 'LTC'<br>11. 'Long-term illness'<br>12. 'Chronic condition'<br>13. 'Physical health'<br>14. 'Long term condition'<br>15. 'Long term illness'
Digestive conditions	16. 'Digestive condition*'<br>17. 'Inflammatory bowel disease'<br>18. 'IBD'<br>19. 'Crohn's'<br>20. 'Reflux'<br>21. 'Stomach ulcer'<br>22. 'Irritable bowel syndrome'<br>23. 'IBS'<br>24. 'Colitis'
Blood disorders	25. 'Blood disorder*'<br>26. 'Sickle cell'<br>27. 'Thalassemia'<br>28. 'Anaemia'<br>29. 'Anemia'<br>30. 'Haemoglobinopath*'<br>31. 'Hemoglobinopath*'<br>32. 'Coeliac'<br>33. 'Celiac'<br>34. 'Haemophilia'<br>35. 'Hemophilia'<br>36. 'Coagulation disorder*'<br>37. 'Hypertension'<br>38. 'Hypotension'
Lung/respiratory conditions	39. 'Lung disease'<br>40. 'Chronic obstructive pulmonary disease'<br>41. 'COPD'<br>42. 'Bronchopulmonary dysplasia'<br>43. 'Cystic fibrosis'<br>44. 'Lung fibrosis'<br>45. 'Obstructive sleep apnoea'
Autoimmune disorders	46. 'Autoimmune disorder*'<br>47. 'Sjögrens'<br>48. 'Lupus'

	49. ‘Multisystem autoimmune disease*’
	50. ‘MSAID’
Neurological/motor disorders	51. ‘Brain injur*’
	52. ‘Stroke’
	53. ‘Transient ischaemic attack’
	54. ‘TIA’
	55. ‘Transient ischemic attack’
	56. ‘Ataxia’
	57. ‘Palsy’
	58. ‘Dyspraxia’
	59. ‘Motor neurone disease’
	60. ‘Muscular dystroph*’
	61. ‘Spina bifida’
	62. ‘Neural tube defect*’
	63. ‘Anencephaly’
	64. ‘Multiple sclerosis’
	65. ‘Epilepsy’
	66. ‘Parkinson's disease’
	67. ‘Dementia’
Heart/circulatory conditions	68. ‘Heart disease’
	69. ‘Cardiac arrhythmia*’
	70. ‘Coronary heart disease’
	71. ‘Angina’
	72. ‘Angioedema’
	73. ‘Heart failure’
	74. ‘Peripheral vascular disease’
	75. ‘Raynaud’s’
	76. ‘Congestive heart failure’
	77. ‘Atrial fibrillation’
Chronic pain conditions	78. ‘Chronic pain’
	79. ‘Migraine’
	80. ‘Cluster headache’
	81. ‘Fibromyalgia’
	82. ‘Gout’
	83. ‘Ankylosing spondylitis’
	84. ‘Arthritis’
	85. ‘Neuralgia*’
	86. ‘Osteoarthritis’
Skin conditions	87. ‘Skin disorder*’
	88. ‘Eczema’
	89. ‘Psoriasis’
	90. ‘Skin condition*’
Endocrine conditions	91. ‘Endocrine disorder*’
	92. ‘Thyrotoxicosis’
	93. ‘Hypogonadism’
	94. ‘Hypothyroidism’
	95. ‘Addison’s disease’
	96. ‘Cushing syndrome’
	97. ‘Thyroid disease’
	98. ‘Diabetes’
	99. ‘Chronic kidney disease’
Gynaecological conditions	100. ‘Gynaecological condition*’
	101. ‘Chronic pelvic pain’
	102. ‘Endometriosis’
	103. ‘Polycystic ovary syndrome’
	104. ‘PCOS’
	105. ‘Polycystic ovary disease’
Other conditions	106. ‘Asthma’
	107. ‘Cancer’

	108. 'Connective tissue disease*'<br>109. 'Chronic fatigue'<br>110. 'Chronic fatigue syndrome'<br>111. 'Osteoporosis'<br>112. 'Ménière's disease'<br>113. 'Chagas disease'<br>114. 'Sarcoidosis'<br>115. 'Incontinence'<br>116. 'Encopresis'<br>117. 'Enuresis'<br>118. 'Allergy'<br>119. 'Myalgic encephalomyelitis'<br>120. 'Human immunodeficiency virus'<br>121. 'HIV'<br>122. 'Hepatitis'

1. 1 OR 2 OR 3 OR 4 OR 5 OR 6 OR 7 OR 8
2. 9 OR 10 OR 11 OR 12 OR 13 OR 14 OR 15
3. 16 OR 17 OR 18 OR 19 OR 20 OR 21 OR 22 OR 23 OR 24
4. 25 OR 26 OR 27 OR 28 OR 29 OR 30 OR 31 OR 32 OR 33 OR 34 OR 35 OR 36 OR 37 OR 38
5. 39 OR 40 OR 42 OR 42 OR 43 OR 44 OR 45
6. 46 OR 47 OR 48 OR 49 OR 50
7. 51 OR 52 OR 53 OR 54 OR 55 OR 56 OR 57 OR 58 OR 59 OR 60 OR 61 OR 62 OR 63 OR 64 OR 65 OR 66 OR 67
8. 68 OR 69 OR 70 OR 71 OR 72 OR 73 OR 74 OR 75 OR 76 OR 77
9. 78 OR 79 OR 80 OR 81 OR 82 OR 83 OR 84 OR 85 OR 86
10. 87 OR 88 OR 89 OR 90
11. 91 OR 92 OR 93 OR 94 OR 95 OR 96 OR 97 OR 98 OR 99
12. 100 OR 101 OR 102 OR 103 OR 104 OR 105
13. 106 OR 107 OR 108 OR 109 OR 110 OR 111 OR 112 OR 113 OR 114 OR 115 OR 116 OR 117 OR 118 OR 119 OR 120 OR 121 OR 122
14. 1 AND 2
15. 1 AND 3 OR 4 OR 5 OR 6 OR 7 OR 8 OR 9 OR 10 OR 11 OR 12 OR 13
